# SOCIAL ISOLATION AS A RISK FACTOR FOR GRIP STRENGTH DECLINE: FINDINGS FROM THE NHATS STUDY

**DOI:** 10.1093/geroni/igad104.2782

**Published:** 2023-12-21

**Authors:** Murad Taani, Julie Ellis, Yura Lee, Chi Cho, Ammar Hammouri, Amy Schlueter

**Affiliations:** University of Wisconsin-Milwaukee, Milwaukee, Wisconsin, United States; University of Wisconsin Milwaukee, Milwaukee, Wisconsin, United States; University of Wisconsin-Milwaukee, Milwaukee, Wisconsin, United States; University of Wisconsin Milwaukee, Milwaukee, Wisconsin, United States; University of Wisconsin Milwaukee, Milwaukee, Wisconsin, United States; Ovation Communities, Milwaukee, Wisconsin, United States

## Abstract

Low grip strength is associated with negative health outcomes among older adults, including poor physical performance, sarcopenia, and all-cause mortality. Social isolation has been also linked with decreased physical function and mortality. Limited studies examined whether social isolation is a risk factor for grip strength decline among older adults and whether race differences exist in this association using population-based longitudinal data. The aim of this study was to evaluate the relationship between social isolation and grip strength among older adults and whether the relationship varies by race and over time. We analyzed National Health and Aging Trends Study (NHATS) data (2015 - 2018) and the sample included 4877 adults > 65 years old. Grip strength was measured using a digital hand dynamometer. Social isolation was measured using six NHATS items from the literature and the composite score ranged from 0 to 6, with a higher score indicating more isolation. The sample mean age was 75 (SD = 22; 68 - 110 years). Repeated-measures analysis of variance showed significant differences in grip strength across social isolation scores (p = .001) indicating that socially isolated older adults have significantly lower grip strength. We also found that grip strength significantly decreased by 2.046 kg over the four-year period (p = < .001). The findings showed no significant difference in grip strength across race and change in grip strength across time did not differ by race and social isolation. Interventions to improve muscle strength and reduce social isolation among older adults are needed.

